# Nivolumab-induced subacute cutaneous lupus erythematosus in a patient with sigmoid colon cancer: a case report and review of the literature

**DOI:** 10.3389/fonc.2025.1674823

**Published:** 2025-11-03

**Authors:** Li Chang, Jianglin Zhang, Zhaojun Sun, Youyou Zhou, Lixiong Zheng

**Affiliations:** Department of Dermatology, Shenzhen People’s Hospital (The First Affiliated Hospital, Southern University of Science and Technology; The Second Clinical Medical College, Jinan University), Shenzhen, Guangdong, China

**Keywords:** nivolumab, immune checkpoint inhibitors, subacute cutaneous lupus erythematosus, drug induced lupus erythematosus, sigmoid colon cancer

## Abstract

**Introduction:**

Immune checkpoint inhibitors (ICIs) are widely used in the treatment of various cancers, but they may lead to multi-system immune-related adverse events (irAEs). Subacute cutaneous lupus erythematosus (SCLE) has rarely been reported, however, it can cause severe adverse cutaneous reactions, which then affect patients’ anti-tumor treatment decisions as well as life and survival. This study presented the first case report of SCLE caused by nivolumab in the patient with sigmoid colon cancer, and summarized the clinical manifestations, diagnostic criteria and specific treatment plans of this type of disease.

**Case presentation:**

After the treatment of sigmoid colon cancer with nivolumab, a 72-year-old male patient developed multiple well-demarcated erythematous annular plaques on the upper trunk and dorsal forearm, with erythematous borders and central clearing. The infiltrating erythema was observed behind the ear and in the occipital area on both sides, with thin scales attached and gradually increasing. Serum laboratory testing indicated an elevated anti-nuclear antibody (ANA) titer, strong positive for Sjogren’s syndrome-A (SS-A)/Ro and Sjogren’s syndrome-B (SS-B) antibodies. The pathological result of skin biopsy suggested SCLE. In terms of treatment, the patient’s rash was significantly improved after suspending the treatment of nivolumab and adding methylprednisolone and hydroxychloroquine. At present, the patient survived and the rash was stable after seventeen months of follow-up.

**Conclusion:**

Through the discussion of this case, the typical characteristics, diagnostic criteria and treatment of DI-SCLE caused by ICIs are summarized in detail, which provides help for early clinical diagnosis and effective management of DI-SCLE caused by ICIs, and effective treatment is expected to prolong the survival period and improve the quality of life of the patients.

## Introduction

Immune checkpoint inhibitors (ICIs) are currently widely used in the treatment of various cancers, but immune-related adverse events (irAEs) caused by ICIs have been frequently reported (1). Nivolumab is an ICI antibody against programmed cell death 1 (PD-1), and a widely used human monoclonal antibody for the treatment of various cancers (2, 3). PD-1 interacts with programmed cell death ligand 1 (PD-L1) and provides inhibitory signals to T cells, which can cause T-cell anergy or exhaustion (2). Tumor cells evade the immune system by expressing PD-L1, suppressing the activity of cytotoxic T cells (3). Therefore, inhibition of PD-1 and PD-L1 interactions may promote an anti-tumor response. However, ICIs may cause irAEs affecting multiple systems, such as skin reactions, enteritis, hepatitis, and arthritis (4). Among them, cutaneous irAEs induced by ICIs have the highest incidence; severe cases of such reactions can affect decisions regarding the patient’s anti-tumor treatment and compromise their prognosis (5, 6). Drug-induced lupus erythematosus (DILE) is a lupus-like syndrome associated with drug exposure that usually resolves after drug withdrawal (7). Cutaneous drug-induced lupus erythematosus (CDILE) may manifest as isolated cutaneous lupus erythematosus (CLE) and is associated with the use of different medications (8). Subacute cutaneous lupus erythematosus (SCLE) is a phenotypic subgroup of CLE between transient acute CLE and chronic cicatricial CLE (9), and is the most common form of DILE (10). Monoclonal antibody therapy, like ICIs, is considered to be one of the reasons for drug-induced subacute cutaneous lupus erythematosus (DI-SCLE) (11).

At present, a small number of studies have reported that ICIs and other monoclonal antibodies can induce SCLE (11, 12), but the data about the causes, clinical features, and pathogenesis of the disease is limited (13). In particular, there are very few reports of DI-SCLE after nivolumab treatment. Therefore, we collected a case of DI-SCLE induced by nivolumab in a patient with sigmoid colon cancer and discussed in detail. It is expected to provide clinicians with valuable data for early identification and timely diagnosis of DI-SCLE, while offering crucial support for cancer patients who require prompt and accurate treatment, as well as long-term monitoring and management. In addition, this is the first reported case of nivolumab-induced SCLE in the patient with sigmoid colon cancer.

## Case presentation

**Figure 1:**
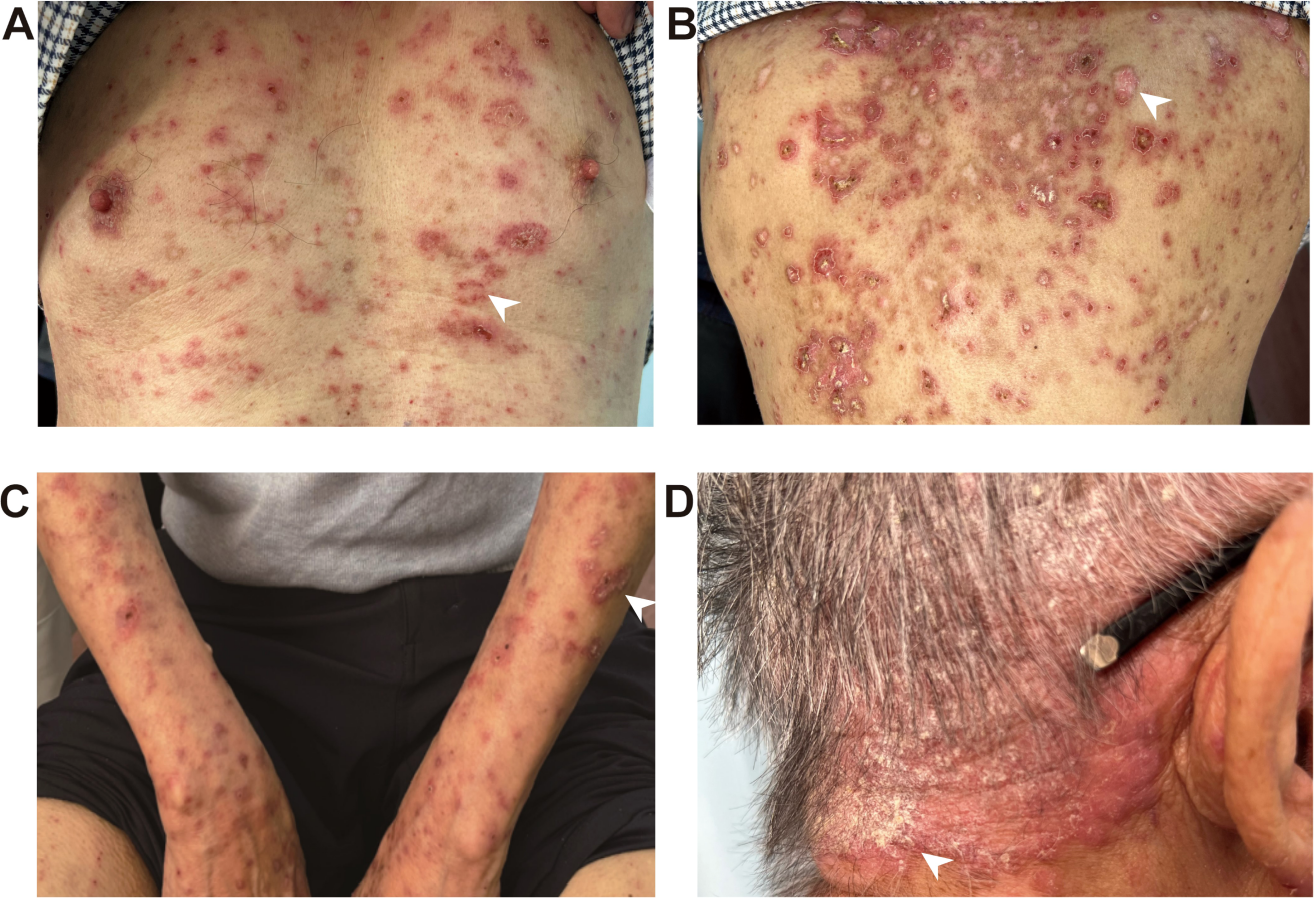
ICI-induced SCLE clinical appearance. Multiple well-demarcated erythematous annular plaques on the upper chest **(A)**, upper back **(B)** and dorsal forearm **(C)**, with erythematous borders and central clearing, as indicated by the white arrows. Infiltrated erythema with fine scaling was observed in the retroauricular region and occipital area, as indicated by the white arrows **(D)**.

A 72-year-old male patient diagnosed with stage IIA sigmoid colon cancer (pT3N0M0) underwent comprehensive genomic profiling (CGP) of tumor tissue taken during a colonoscopy, including analysis of KRAS, NRAS, BRAF, MMR/MSI, and other important biomarkers. CGP was performed under the institutional clinical trial protocol and the patient’s request. The results demonstrated a dMMR/MSI-H phenotype, while no mutations were detected in the other biomarkers including KRAS, NRAS, and BRAF. Furthermore, due to the patient’s large-volume tumor causing partial intestinal obstruction and the strong preference of the patient and his family for minimal surgical trauma, medical oncology decided to administer individualized neoadjuvant nivolumab immunotherapy to the patient prior to surgery, following multidisciplinary discussions with gastrointestinal surgery and under direct oncologic supervision. Although the use of neoadjuvant nivolumab in resectable stage IIa (dMMR/MSI-H) colon cancer is off-label and not recommended by NCCN v4.2025, the decision in this case was an individualized off-label choice made based on the patient’s condition and request. It has also been confirmed that the patient’s written informed consent was obtained, and the treatment was administered under ethical supervision. In additon, therapeutic selection of nivolumab was based solely on the dMMR/MSI-H phenotype, and that KRAS/NRAS/BRAF results were incidental and had no impact on management. Consequently, the patient received the first dose of nivolumab (480mg every 4 weeks) on December 31, 2022, followed by a second dose on February 1, 2023. Complete surgical resection of the sigmoid colon tumor was subsequently performed in March, 2023. No adjuvant chemotherapy or additional antitumor therapy was administered postoperatively. Two months post the second nivolumab administration, the patient developed a red rash on the trunk, prompting the discontinuation of nivolumab therapy. By the seventh month, the erythema had significantly worsened, and there was no notable improvement despite the application of both oral and topical traditional Chinese medicine. After ten months, the patient presented with erythema and papules located on the scalp, retroauricular regions, trunk, and extremities, accompanied by pruritus. Notably, there were no other adverse effects associated with nivolumab reported and the patient exhibited no symptoms of mucosal ulceration, joint swelling, pain, or renal involvement. The patient subsequently sought evaluation at the dermatology clinic of our institution. The patient’s medical history included sigmoid colon cancer for over two years and eczema persisting for more than 20 years; he had no known history of autoimmune diseases, prolonged ultraviolet (UV) exposure, or family history. Since initiating nivolumab treatment, he had not introduced any new medications into his regimen and denied taking any known photosensitizing agents. Upon physical examination, multiple well-demarcated erythematous annular plaques on the upper trunk and dorsal forearm, with erythematous borders and central clearing (**Figures 1A–C**). Notably, infiltrated erythema with fine scaling was observed in the retroauricular region and occipital area (**Figure 1D**).

**Figure 2:**
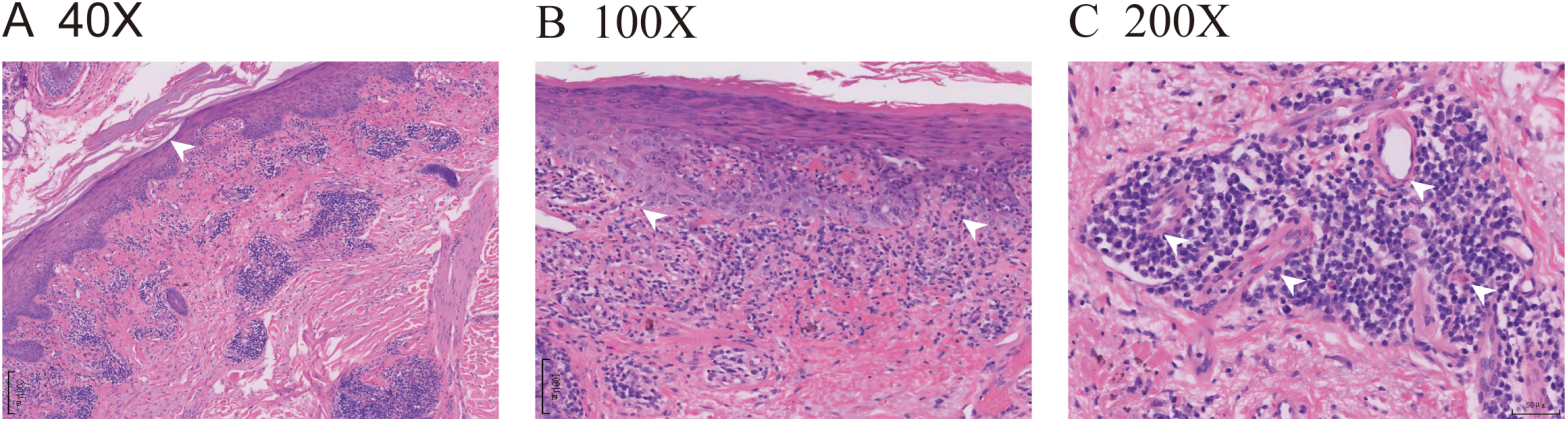
ICI-induced SCLE histopathologic appearance. **(A)** (HE 40X) Histopathologic sections of a hematoxylin and eosin-stained skin biopsy revealed epidermal hyperkeratosis with areas of hypokeratosis, epithelial angle down-extension, as indicated by the white arrow. **(B)** (HE 100X) Dermal fibrous tissue had hyperplasia with collagenization, and focal interface dermatitis changes were observed at the dermoepidermal junction, as indicated by the white arrows. **(C)** (HE 200X) Lymphocytes and plasma cells were visibly infiltrated around the small blood vessels, as indicated by the white arrows.

**Figure 3:**
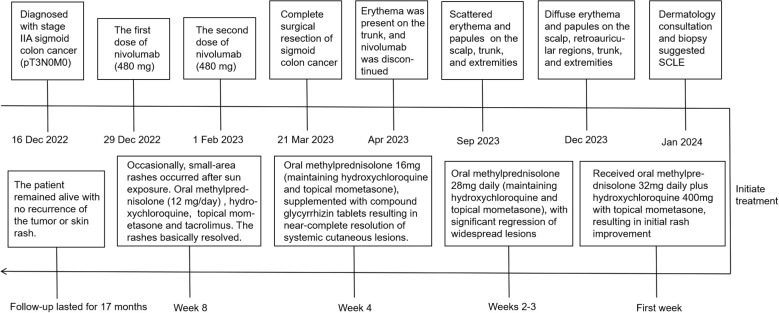
Timeline of the patient’s treatment course.

The pathological findings from the skin biopsy revealed epidermal hyperkeratosis with areas of hypokeratosis, downward extension of the epithelial rete ridges, dermal fibrous tissue hyperplasia with collagenization, focal interface dermatitis, and significant infiltration of lymphocytes and plasma cells surrounding small blood vessels (**Figures 2A–C**). Serum laboratory testing indicated an elevated anti-nuclear antibody (ANA) titer ranging from 1:100 to 1:320 with a granular pattern. Notably, Sjögren’s syndrome-A (SS-A)/Ro and Sjögren’s syndrome-B (SS-B) antibodies were strongly positive. Anti-histone antibodies and anti-dsDNA antibodies were negative. Tests for the following autoantibodies all yielded negative results: anti-ribonucleoprotein, anti-smith, anti-Scl-70, anti-PM-Scl, anti-Jo-1, anti-centromere, anti-histone, anti-ribosomal p protein, and anti-nucleosome antibodies. Additionally, the test for proliferating cell nuclear antigen (PCNA) was also negative. The detected levels of immunoglobulin A, G, and M, as well as complement factor 3 (C3) and factor 4 (C4), were all within the normal reference ranges. Furthermore, the complete blood count (CBC) revealed pancytopenia, with a white blood cell (WBC) count of 3.26×10^9^/L (normal range: 3.5-9.5×10^9^/L), hemoglobin (Hb) of 103 g/L (normal range: 130–175 g/L), and platelet (PLT) count of 114×10^9^/L (normal range: 125-350×10^9^/L). The erythrocyte sedimentation rate (ESR) was elevated, measuring 53 mm/h (normal range: 0–15 mm/h). No abnormalities were observed in coagulation function, electrolytes, liver function, or renal function, and no systemic involvement was identified in the patient. The diagnosis of DI-SCLE associated with nivolumab therapy was established based on the patient’s clinical manifestations, cutaneous histopathology, and serological findings. The use of nivolumab was suspended. During the first week, the patient received oral methylprednisolone 32mg daily plus hydroxychloroquine 400mg with topical mometasone, resulting in initial rash improvement. Subsequently, the oral dose of methylprednisolone was gradually tapered, with the specific adjustment regimen shown in **Figure 3**. By Week 4, the patient achieved nearly complete resolution of generalized cutaneous lesions. At Week 8, the patient occasionally developed small, localized new skin lesions following sun exposure. Oral methylprednisolone (12 mg/day) and hydroxychloroquine (400 mg/day) were continued, along with topical mometasone and tacrolimus ointment. With this treatment regimen, the patient’s rash resolved within approximately one week. Additionally, the patient’s cancer progression was actively monitored. Throughout the 17-month follow-up period, the patient exhibited no recurrence of skin rash, no evidence of tumor recurrence on regular monitoring (e.g., tumor markers and contrast-enhanced abdominal CT), and maintained overall stable condition.

## Discussion

DILE refers to a class of clinical syndromes with similar clinical manifestations and serological characteristics of systemic lupus erythematosus after taking certain drugs (14). Different drugs cause different adverse reactions, and many drugs have been found to be involved in causing DI-SCLE, including angiotensin-converting enzyme inhibitors, thiazide diuretics, proton pump inhibitors (PPIs), ICIs, antimicrobials (11, 13, 15). ICIs act through non-specific activation of the immune system and thus induce similar autoimmune diseases, most commonly affecting organs such as the skin, endocrine system, and liver (16).

Nivolumab is a fully human IgG4 PD-1 immune-checkpoint-inhibitor antibody (17). The binding of endogenous PD-1 to one of its two ligands, PD-L1 and PD-L2, generates inhibitory signals, leading to the suppression of T cell activation, proliferation, cytokine production, and reduction of cytotoxic activity, thereby significantly weakening the immune response (18). Nivolumab selectively blocks receptor activation of PD-L1 and PD-L2, leading to the release of PD-1-mediated suppression of the immune response (19). At present, nivolumab, with certain safety and tolerability, is used to treat patients with renal cell carcinoma, metastatic melanoma, colorectal cancer, non-small cell lung cancer and so on (20). To identify high-risk subgroups among patients with colorectal cancer at an early stage, molecular testing was performed on this patient, which will help refine prognostic assessment, predict potential metastatic risk, and guide the direction of precision therapy for the patient (21). This is critical for enabling early intervention and will further contribute to improved overall survival (OS) (22). The results demonstrated a dMMR/MSI-H phenotype. Cancers with dMMR/MSI-H are characterized by elevated tumor mutational burden (TMB), increased production of tumor-derived neoantigens, and more robust intratumoral T-cell infiltration (23, 24). Studies have identified strong expression of immune checkpoint ligands in this type of tumor microenvironment, including PD-1 (25), which accounts for the higher sensitivity and superior efficacy of PD-1 inhibitors in dMMR/MSI-H tumors (26). Therefore, nivolumab was selected as the treatment for the patient reported in our study. However, recent studies have reported that patients with non-small cell lung cancer, melanoma, and gastroesophageal junction adenocarcinoma have caused DI-SCLE after the use of nivolumab (3, 27–29). In addition to SCLE, nivolumab can also cause bullous lupus, vitiligo, lichenoid skin, and other adverse reactions (12).

IrAEs can occur at any time during treatment or several months after stopping treatment (1), but DILE generally develops a few weeks to several months after drug exposure (14). Our patient developed skin symptoms of SCLE about 4 months after starting nivolumab treatment. Studies have reported that even after discontinuation of nivolumab treatment, the drug’s effects may persist from 16 weeks to over 56 weeks due to prolonged saturation of PD-1 receptors for several months post-treatment (3, 30). Furthermore, as ICIs targeting the PD-1 pathway are designed to reduce immune tolerance and reverse T-cell exhaustion in malignancies, the loss of inhibitory signals transmitted through these pathways may shift the balance toward T-cell-mediated inflammation and disruption of self-tolerance (31). Additionally, individual variations in genetic susceptibility, hormonal profiles, inflammatory status, and systemic involvement lead to differential degrees and temporal patterns of immune tolerance breakdown, consequently resulting in heterogeneous timelines for the development of autoimmune manifestations (32).

The clinical manifestations and histopathological features of DI-SCLE are similar to those of LE, with erythema, ring-shaped, papulosquamous lesions, mainly located in sun-exposed areas such as the neck, shoulders, upper chest, arms (with a “V” shaped distribution), and upper back (33); however, they can also occasionally be observed in non-sun-exposed areas (3). The typical histopathological pattern is interfacial dermatitis with lichenoid infiltration, which is characterized by lymphocytes confined to the upper dermis surrounding blood vessels, epidermal thinning, abnormal keratinized cells extending to the upper spine, and basal cell vacuolation (34).

**Figure 4:**
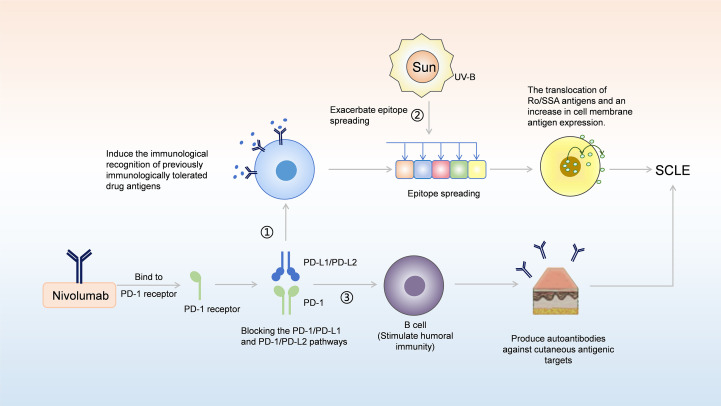
A model for potential involvement of nivolumab in the pathogenesis of SCLE. (1) By blocking the PD-1/PD-L1 and PD-1/PD-L2 pathway, nivolumab can induce the immunological recognition of previously immunologically tolerated drug antigens, which in turn leads to epitope spreading; this spreading promotes the translocation of Ro/SSA antigens and the increase in cell membrane antigen expression, thereby contributing to the development of SCLE. (2) Stimulation by UV-B radiation can further exacerbate epitope spreading; by promoting the translocation of Ro/SSA antigens and increasing the expression of cell membrane antigens, it ultimately leads to the development of SCLE. (3) PD-1/PD-L1 and PD-1/PD-L2 blockades can directly stimulate B cell mediated humoral immunity, resulting in the production of autoantibodies against cutaneous antigenic targets, thereby facilitating the onset of SCLE.

The causes of nivolumab-induced SCLE may be related to the genetic susceptibility, drug conversion, and epigenetic disorders of the body (35), and may also be related to the systemic activation of the immune system caused by nivolumab (29). In our case, the potential mechanism by which nivolumab induces SCLE is illustrated in **Figure 4**, which can be explained in conjunction with the presence of positive autoimmune antibodies. In DI-SCLE, especially ICIs-induced SCLE, the main serum laboratory feature is that most patients are positive for ANA and Ro/SSA antibodies (36). Circulating antibodies Ro/SSA ribonucleoprotein antigen strongly supports the diagnosis of subacute CDILE (7), with Ro/SSA antibodies being positive in up to 80% of cases. La/SSB antibodies are positive in 25% of cases (37). These are consistent with the positive ANA, Ro/SSA, and SSB antibodies in our case. Approximately one-third of DI-SCLE cases test positive for anti-histone antibodies (38). The positivity of autoantibodies may be related to the formation of immune complexes, which is driven by UV-B radiation promoting the translocation of Ro/SSA antigens and increased exposure of autoantigens on the cell surface; this process subsequently leads to antibody-dependent cell-mediated cytotoxicity (29, 39). Anti-double-stranded DNA (anti-dsDNA antibodies) are rare in DILE, and complement levels are usually normal (7). The prevalence of anti-dsDNA antibodies in monoclonal antibody induced SCLE is higher than that of DI-SCLE (40). However, the anti-dsDNA antibody in our case was negative, which is consistent with other case reports of ICIs-induced SCLE (29, 41). As reported in the literature, PD-1/PD-L1 inhibitors may modulate humoral immunity, thereby amplifying pre-existing autoantibodies and unmasking subclinical autoimmunity (42). PD-1/PD-L1 and PD-1/PD-L2 pathway, nivolumab can induce the immunological recognition of previously immunologically tolerated drug antigens, which in turn leads to epitope spreading (43). This spreading promotes the translocation of Ro/SSA antigens and an increase in cell membrane antigen expression, thereby contributing to the development of SCLE (42, 43). As with many other autoimmune phenomena, the presence of autoantibodies indicates B-cell responsiveness to immunotherapy (44). Moreover, direct stimulation of B-cell-mediated humoral immunity by ICIs can generate autoantibodies targeting cutaneous antigens (42, 45). However, interindividual variability in anti-dsDNA antibody expression exists due to the involvement of genetic predisposition, drug biotransformation, and epigenetic dysregulation across immune cell subsets (39). Nevertheless, this does not affect the final diagnosis of SCLE, as the diagnosis is determined comprehensively based on characteristic cutaneous lesion morphology, laboratory findings, histopathological features, differential diagnosis, therapeutic response, and other relevant factors (46).

The diagnosis of ICI-induced SCLE can be challenging, and it is crucial to differentiate between paraneoplastic SCLE and idiopathic SCLE; detailed differentiation criteria are provided in **Table 1**. The diagnostic criteria for paraneoplastic SCLE were established over 30 years ago, requiring that SCLE must manifest after the onset of cancer and that both conditions must progress in parallel (51). On average, paraneoplastic SCLE develops 6 months after cancer diagnosis (range: 0–48 months), resolves during cancer remission, and reappears if the malignancy recurs (52). However, in our case, the patient developed SCLE 4 months after cancer diagnosis and 1 month after tumor resection. The mean age of onset for idiopathic SCLE is 54 years, whereas PD-1/PD-L1 inhibitor-induced SCLE typically presents at an older age (53). While idiopathic SCLE predominantly affects sun-exposed areas, PD-1/PD-L1 inhibitor-associated SCLE may additionally involve non-sun-exposed regions (3). Furthermore, systemic symptoms and signs-particularly arthralgia, arthritis, dry eyes, or nephropathy that frequently accompany idiopathic SCLE-are generally absent in PD-1/PD-L1 inhibitor-induced cases (39). The patient we reported presented with cutaneous manifestations alone, without any systemic involvement.

**Table 1:** Clinical manifestations and management of nivolumab-induced SCLE in the literature.

Patient No./ Sex/Age, y (References)	Tumor type	ICI to SCLE onset (cycles)	Cutaneous eruption sites	Morphological characteristics	Positive autoantibody serologies	ICI management	SCLE treatment	SCLE response	Tumor response
1/F 54y (55, 56)	NSCLC	3.25 mo (NR)	Cheeks, nasal bridge, on the V-neck of the chest, the upper back, the upper outer arms, forearms and legs	Darkly erythematous to violaceous patches and thin scaling annular plaques	ANA, Anti-Ro/SSA, Anti-Ro/SSB	Discontinuation	Topical and systemic CS, HCQ	Improvement	NR
2/F/54y (57)	MSCLC	20 mo (NR)	Trunk and extremities	Annular eruption	ANA, Anti-Ro/SSA, Anti-Ro/SSB	Continuation	Topical CS, HCQ	Resolution	NR
3/M/60y (57)	MM	0.5 mo (NR)	Trunk and extremities	Annular eruption	ANA, Anti-Ro/SSA	Continuation	Topical CS	Improvement	NR
4/M/60y (28)	MSCLC	1 mo (2)	Photo-distributed	Erythematous macules and scaly papules	ANA, Anti-Ro antibody	Rechallenge	Topical and systemic CS, HCQ	Resolution	DP
5/F/58y (27)	MNSCLC	5 mo (NR)	Mainly involving the trunk	Polycyclic and annular papulosquamous plaques	Anti-Ro52/Ro60	Rechallenge	Topical and systemic CS, HCQ	Improvement	NR
6/M/75y (58)	MNSCLC	2.5 mo (5)	Chest, back, legs, arms, dorsal aspect of the hands, and cheeks	Erythematous eruption and papulosquamous plaques with an annular configuration	ANA, Anti-SSA/Ro60	Discontinuation	Systemic CS	Improvement	DP
7/F/66y (58)	NSCLC	12 mo (9)	Trunk, limbs and face	Annular papulosquamous and crusted plaques	ANA, Anti-SSA/Ro60	Rechallenge	Systemic CS	Resolution	DP
8/F/72y (3)	SSM	2 mo (post cycle 13)	Back and arms	Nummular erythematous plaques	ANA, Anti-Ro/SSA, Anti-La/SSB	Discontinuation	Topical CS, HCQ	Improvement	CR
9/M/43y (3)	NSCLC	1 mo (2)	Hands, arms, chest, and the rest of the body	annular erythematous eruption	ANA Anti-Ro/SSA	Discontinuation	Topical and systemic CS, HCQ	Improvement	DP
10/M/75y (12)	GJA	1 mo (1)	Bilateral dorsal forearms, upper arms, upper chest, and upper back	Photo-distributed oval pink to violaceous scaly papules and plaques	ANA	Discontinuation	Topical and systemic CS, HCQ	Improvement	DP

SCLE, subacute cutaneous lupus erythematosus; ICI, immune checkpoint inhibitor; y, year; F, female; M, male; NSCLC, non–small cell lung cancer; mo, months; ANA, antinuclear antibody; SSA, Sjögren’s syndrome-A; SSB, Sjögren’s syndrome-B; CS, corticosteroids; HCQ, hydroxychloroquine; NR, not reported; CR, complete response; DP, disease progression; MSCLC, metastatic small cell lung carcinoma; MM, metastatic melanoma; MNSCLC, metastatic non-small cell lung cancer; SSM, superficial spreading melanoma; GJA, gastroesophageal junction adenocarcinoma.

Treatment of ICI-induced SCLE depends on several factors, including severity of rash, treatment response, and the setting in which ICIs are treated (54). Through a comprehensive review of currently reported cases of nivolumab-induced SCLE, we have systematically summarized the clinical manifestations and treatment strategies for this condition (**Table 2**). Furthermore, severity of rash is an important indicator for us to formulate treatment plans and is graded according to the Common Terminology Criteria for Adverse Events (59). Patients with grade 1–2 toxicity (<30% body surface area (BSA), with or without mild symptoms), are treated with moderate-high potency topical corticosteroids. Systemic corticosteroids (prednisone 0.5–1 mg/kg daily, tapered over 4 weeks) may also be necessary. Patients with grade 3 toxicity (>30% BSA, moderate-severe symptoms), require withholding of ICIs treatment, dermatology consultation, and systemic corticosteroids (prednisone 1 mg/kg daily, tapered over at least 4 weeks). Grade 4 toxicity (requiring urgent intervention or hospitalization) should be treated with IV methylprednisolone 1–2 mg/kg daily with slow tapering once the toxicity resolves and necessitates reconsideration of ICIs rechallenge (29, 60). In addition, other adjuvant medications can be incorporated to help improve rashes and repair the skin barrier. In our case, the patient was treated with compound glycyrrhizin, which also contributed to the improvement of the skin condition. Compound glycyrrhizin (CG) contains glycyrrhizin, aminoacetic acid, and methionine (61), and exhibits anti-inflammatory, anti-allergic, and immunomodulatory effects. It can exert glucocorticoid-like effects safely (62). In China, it is an immunomodulator commonly used in the treatment of immunological skin conditions (63). In addition, after the occurrence of DI-SCLE by ICIs, close follow-up is necessary if immunotherapy is continued. In the case of nivolumab induced SCLE reported by Marano et al, the patient continued nivolumab treatment after the rash recovered, and then it progressed to the anti-melanoma differentiation-associated gene 5 (anti-MDA-5) antibody associated dermatomyositis (28). Although nivolumab was suspended later, unfortunately, the patient died from the worsening of the disease.

**Table 2:** Differential diagnosis of ICI-induced SCLE, paraneoplastic SCLE, and idiopathic SCLE.

Distinguishing features/Diseases	ICI-induced SCLE	Paraneoplastic SCLE	Idiopathic SCLE
Incubation period	4 to 14 months after drug administration, or even more than 14 months.	An average of 6 months (range: 0–48 months) after cancer diagnosis.	No incubation period.
Diagnostic criteria	(A) one or more clinical symptoms consistent with SCLE; (B) no history of SLE prior to use of this specific drug; (C) sufficient and sustained exposure to a specific drug; (D) the symptoms will be relieved when the specific drug is discontinued; (E) systemic symptoms and signs rarely occur (47).	(A) cutaneous lesions of SCLE occur subsequent to the onset of malignant tumors; (B) the skin disease and malignant tumor exhibit a parallel progression trend; (C) the rash should resolve following the treatment of malignant tumors and recur upon tumor relapse.	(A) typical cutaneous lesions of SCLE; (B) systemic symptoms and signs are often present, particularly arthralgia, arthritis, dry eyes, or nephropathy; (C) other known secondary factors (e.g., drugs, malignant tumors) are excluded.
Clinical features	Erythema, ring-shaped lesions, and papulosquamous lesions are mainly located in sun-exposed areas, but they can also involve non-sun-exposed sites and are rarely accompanied by systemic symptoms.	Erythematous, papulosquamous/psori- asiform, or annular lesions can occur in both sun-exposed and non-sun-exposed areas, and they are rarely accompanied by systemic symptoms (48, 49).	The typical photosensitive symmetric, nonscarring annular polycyclic or papulosquamous lesions, usually occur in sun-exposed areas (50), often accompanied by systemic symptoms and signs.
Histopathological pattern and immunofluorescence	Interface dermatitis and perivascular lymphocytic infiltration in the dermis. Granular deposition of IgM, IgG and C3 in a band-like array at the dermal-epidermal junction, and the direct immunofluorescence findings are most commonly described as granular staining at the dermal-epidermal junction (38).	Interface dermatitis and perivascular lymphocytic infiltration in the dermis. Immune complex depositionin can be scant or negative (49).	Focal vacuolization of the epidermal basal layer associated with a perivascular dermal lymphocytic infiltrate (38). Granular deposition of IgM, IgG and C3 in a band-like array at the dermal-epidermal junction, and the direct immunofluorescence findings are most commonly described as granular staining at the dermal-epidermal junction (38).
Laboratory features	Most patients are positive for ANA and Ro/SSA antibodies. La/SSB antibodies are positive in 25% of cases.	A high frequency of ANA and Ro/SSA antibodies and a low frequency of La/SSB antibodies (48).	The frequent presence of anti-Ro/SSA and/or anti-La/SSB, together with ANA (50).

IgM, immunoglobulin A; IgG, immunoglobulin G; C3, complement factor 3; other abbreviations are provided in the Notes of **Table 1**.

Our case report provides clinically relevant data that may assist physicians in both early identification and timely diagnosis of ICI-induced SCLE, while simultaneously facilitating appropriate management for cancer patients requiring prompt and accurate treatment. Furthermore, these findings underscore the importance of enhanced dermatological surveillance and long-term monitoring for cutaneous manifestations in patients undergoing ICI therapy. Given that approximately 50% of SCLE cases meet the American College of Rheumatology (ACR) classification criteria for systemic lupus erythematosus, patients receiving ICIs require regular monitoring for organ involvement, disease activity, cutaneous manifestations, and quality of life (64). As well as educating the patient on sun protection and avoiding prolonged UV exposure, which is crucial for reducing rash recurrence (29). When clinically indicated, rheumatologic serologic tests should be performed (36). For patients diagnosed with ICI-induced SCLE, disease activity and skin lesion severity must be evaluated at initial onset and monitored periodically during follow-up.

Furthermore, significant research gaps remain that warrant further investigation, particularly in developing biomarkers to predict the risk of SCLE induced by ICIs such as nivolumab, and in establishing the prevalence and characteristic patterns of case-specific autoantibody profiles in this disease entity (39). Additionally, while nivolumab is applicable for treating various cancer treatments (20, 65), as shown in **Table 2**, documented cases of nivolumab-induced SCLE have primarily been reported in small cell lung cancer, non-small cell lung cancer, and melanoma, with pulmonary malignancies constituting the majority (7 out of 10 cases). These findings suggest a potential association between nivolumab-induced SCLE and specific tumor types, possibly due to differences in tumor immunogenicity or patient-specific immune responses. Notably, previous studies have found that SCLE is associated with various malignancies in patients older than 50 years, including lung, breast, gastric, uterine cancers, melanoma and so on (66). However, the number of reported cases of nivolumab-induced SCLE remains limited, making it difficult to establish a definitive correlation. Therefore, future research should place greater emphasis on nivolumab-induced SCLE. By increasing case reports, further studies can be conducted to determine whether specific tumor types predispose patients to this complication.

To sum up, reports on nivolumab-induced SCLE or ICI-induced SCLE remain extremely limited in the medical literature, and there is a lack of systematic and comprehensive review studies. This study provides a relatively thorough summary of the clinical manifestations, histopathological features, laboratory findings, disease severity, and corresponding treatment strategies for ICI-induced SCLE, with a focus on nivolumab as a representative agent. Furthermore, we propose specific long-term monitoring and follow-up protocols for patients receiving ICIs and discuss the impact of ICI-induced SCLE on treatment continuation and oncological outcomes. These findings aim to improve the diagnosis and management of ICI-induced SCLE. Multidisciplinary research into the skin toxicity of these drugs facilitates early diagnosis and effective management, which allows patients to continue to receive some of the treatments that prolong survival, as shown in this case.

### Study limitations

We reported only one case to analyze the characteristics, diagnosis and treatment of DI-SCLE caused by ICIs, which limited our study to some extent. Due to the rarity of nivolumab-induced SCLE, the number of cases is very limited, so we analyzed and summarized this disease on the basis of existing research. Moreover, during the initial visit and follow-up periods, our monitoring primarily focused on cutaneous manifestations and systemic symptoms of ICI-induced SCLE, as well as tumor progression status, while the assessment of disease activity remained incomplete. This underscores the necessity for implementing more comprehensive evaluations of disease activity in the clinical management and follow-up of patients with ICI-induced SCLE. Such dynamic and holistic disease assessments would enable optimization of therapeutic strategies. In addition, our follow-up time was not long enough, and the long-term prognosis of the patient also required further follow-up in the future.

## Conclusion

In summary, our report suggests that the use of ICIs may cause SCLE, and that early diagnosis and treatment are important for prognosis. By providing a detailed overview of the typical features and diagnostic criteria of ICI-induced SCLE, clinicians can improve their understanding of ICI-induced SCLE diagnosis and treatment, avoiding unnecessary tests and speeding up appropriate treatment. Multidisciplinary research into the skin toxicity of these drugs facilitates early diagnosis and effective management, which allows patients to continue to receive these life-prolongation treatments. With the increasing application of ICIs, while bringing medical progress, we should also pay attention to the adverse reactions it may cause, such as SCLE, and then diagnose and treat in time. In addition, although this is a case report, rare disease case reports and summaries of diagnosis and treatment are important and will provide a basis for future research.

## Data Availability

The original contributions presented in the study are included in the article/Supplementary Material. Further inquiries can be directed to the corresponding author.
